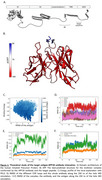# *In silico* characterization of a novel antibody against the C‐terminal intracellular region of the amyloid precursor protein

**DOI:** 10.1002/alz70855_102604

**Published:** 2025-12-23

**Authors:** Érika Sánchez‐Aced, Sergi Roda, Sonia Sirisi Dolcet, Victor Guallar, Alberto Lleó

**Affiliations:** ^1^ Center for Biomedical Investigation Network for Neurodegenerative Diseases (CIBERNED), Madrid, Spain; ^2^ Sant Pau Memory Unit, Hospital de la Santa Creu i Sant Pau, Institut de Recerca Sant Pau ‐ Universitat Autònoma de Barcelona, Barcelona, Spain; ^3^ Nostrum Biodiscovery S.L., Protein Engineering Department, Barcelona, Spain; ^4^ Barcelona Supercomputing Center (BSC), Barcelona, Spain; ^5^ CIBERNED, Network Center for Biomedical Research in Neurodegenerative Diseases, National Institute of Health Carlos III, Madrid, Spain

## Abstract

**Background:**

The characterization of the C‐terminal intracellular region of the amyloid precursor protein (APP) has been challenging due to its intrinsically disordered nature. Unlike the E1 and E2 subdomains, which show well‐defined folded structures (Figure 1A), the C‐terminal region does not adopt a fixed conformation under normal physiological conditions. Furthermore, while this domain can adopt specific folds upon binding to certain interactors, its structural flexibility and lack of complete structural information complicates the development of specific antibodies against this region. Here, we generated and characterized a novel monoclonal antibody (APP1B) against the C‐terminal region of APP outside of the Ab region and performed structural computational analyses of the antibody.

**Method:**

The antibody, antigen, and antibody‐antigen structures were predicted using AlphaFold2.3 and AlphaFold2.3 Multimer. The obtained structures were used to predict the specificity of the developed antibody against the target peptide (by comparing the confidence scores against the other soluble peptide fragments of the APP protein), and computationally predict its binding affinity against the antigen using Protein Energy Landscape Exploration (PELE) and Molecular Dynamics (MD).

**Result:**

The antigen adopted a fixed, high‐confidence structure in the presence of the antibody, revealing a clear binding site (Figure 1B). Using PELE, we identified an energetic minimum at ∼6 Å from the initial antigen‐antibody conformation, with the antibody's interacting region remaining stable. The solvent‐accessible surface area analysis indicated reduced solvent accessibility for the antigen due to antibody binding (Figure 1C). MD simulations showed high RMSD variability in the antibody's heavy chain CDR3, while the antigen's targeted region exhibited lower, stable RMSD than the whole antigen, confirming interaction with the antibody paratope (Figure 1D‐F). Specificity analysis using AlphaFold2 Multimer revealed the highest predicted alignment error (pAE) at the APP1B‐target interface compared to other APP fragments, and no significant interactions with APLP1 or APLP2, confirming antibody specificity.

**Conclusion:**

Our computational and structural analyses validate APP1B's high‐affinity interaction with the target antigen (APP C‐terminal region). This specificity, coupled with the lack of cross‐reactivity with APLP1 or APLP2, positions APP1B as a promising candidate for targeted research.